# Maternal immune suppression during pregnancy does not prevent abnormal behavior in offspring

**DOI:** 10.1186/s13293-024-00600-8

**Published:** 2024-03-26

**Authors:** Ashley Griffin, Teylor Bowles, Lucia Solis, Teryn Railey, Samer Beauti, Reanna Robinson, Shauna-Kay Spencer, James P Shaffery, Kedra Wallace

**Affiliations:** 1 Program in Neuroscience, University of Mississippi Medical Center, 2500 North State Street, Jackson, MS 39216 USA; 2 Department of Pharmacology & Toxicology, University of Mississippi Medical Center, 2500 North State Street, Jackson, MS 39216 USA; 3 Department of Obstetrics & Gynecology, University of Mississippi Medical Center, 2500 North State Street, Jackson, MS 39216 USA; 4 Master’s in Biomedical Science program, School of Graduate Studies in Health Sciences, University of Mississippi Medical Center, 2500 North State Street, Jackson, MS 39216 USA; 5 Department of Psychiatry and Human Behavior, University of Mississippi Medical Center, 2500 North State Street, Jackson, MS 39216 USA

**Keywords:** sFlt-1, Preeclampsia, HELLP syndrome, ADHD, Neurodevelopment, Hypertensive disorders of pregnancy, Offspring behavior, Sex differences

## Abstract

**Background:**

Offspring of hypertensive disorders of pregnancy are at an increased risk of developing neurodevelopmental and neurobehavioral disorders compared to offspring from non-affected pregnancies. Using rodent models of Preeclampsia (PreE; new onset of hypertension after 20 weeks gestation) and HELLP (hemolysis, elevated liver enzymes, and low platelets), we studied the behavioral outcome of their offspring in adolescence.

**Methods:**

A subset of dams received Orencia, a T-cell activation inhibitor, as T cells have been associated with the induction of hypertension and inflammation during pregnancy. We hypothesized that offspring from hypertensive dams would experience adverse behavioral outcomes in social, cognitive, locomotor, and anxiety tests, and offspring from dams treated with Orencia would demonstrate less adverse behaviors.

**Results:**

Male offspring of PreE + Orencia dams (*p* < 0.05) and female offspring from HELLP + Orencia dams (*p* < 0.05) spent more time playing compared to normal pregnant offspring. All offspring from hypertensive and Orencia-treated dams performed worse on the Barnes Maze test compared to normal pregnant. We also measured adult (postnatal day > 60) myelin basic protein (MBP) and NeuN expression in both the prefrontal cortex and hippocampus. In the hippocampus and prefrontal cortex, there was no difference in expression of either MBP or NeuN in all groups regardless of sex.

**Conclusion:**

The results from this study suggest that offspring of hypertensive disorders of pregnancy have behavioral changes, specifically cognitive differences. This study has shown that there is a sex dependent difference in offspring neurobehavioral development, influenced in part by the type of hypertensive disorder of pregnancy, and alterations in the maternal immune system.

**Supplementary Information:**

The online version contains supplementary material available at 10.1186/s13293-024-00600-8.

## Highlights

Female offspring from HELLP dams treated with or without Orencia (Abatacept, prevents T cell activation) have decreased locomotion.

Male offspring from PreE dams and females from HELLP dams that have been treated with Orencia have increased social play.

Offspring of both hypertensive and Orencia treated dams have spatial memory deficits.

Male offspring from PreE dams treated with Orencia show evidence of anxiety-like behavior in marble burying tasks.

**Supplementary Information:**

The online version contains supplementary material available at 10.1186/s13293-024-00600-8.

## Plain language summary

Children of pregnancies that are complicated by hypertensive disorders of pregnancy (HDP) such as Preeclampsia and HELLP (hemolysis, elevated liver enzymes, and low platelets) syndrome have an increased risk of having behavioral deficits and changes in brain development. Both Preeclampsia and HELLP have been shown to activate the immune and inflammation systems in the body of the mother. In this study, we used offspring of rat models of Preeclampsia and HELLP to study their behavior including anxiety-like behaviors and memory deficits. We also compared offspring of rat models of Preeclampsia and HELLP that were given Orencia, which minimizes immune responses by blocking the activation of T cells. We also studied two regions of the brain (prefrontal cortex and hippocampus) to measure two proteins (myelin basic protein (MBP) and NeuN) involved in brain function. Our study found that offspring from dams that were treated with Orencia during pregnancy with HDP had sex differences in time playing. All offspring, regardless of the HDP dam being treated with or without Orencia, had evidence of spatial learning deficits. When sexes and groups were compared there was no difference in MBP or NeuN expression in the prefrontal cortex or hippocampus.

**Supplementary Information:**

The online version contains supplementary material available at 10.1186/s13293-024-00600-8.

## Introduction

Maternal cardiovascular disorders during pregnancy have long-term effects on offspring that can range from developmental to metabolic disorders [1]. Hypertensive disorders of pregnancy (HDP) such as Preeclampsia (PreE, new onset of both hypertension and proteinuria presenting after 20 weeks gestation) and HELLP (hemolysis, elevated liver enzymes, and low platelets) affect approximately 3–8% of pregnancies worldwide [2, 3]. The most commonly accepted pathophysiological mechanism for PreE and HELLP is that inadequate invasion of the uterine spiral arteries leads to abnormal oxygenation of the placenta followed by an imbalance of angiogenic factors [4]. Currently the treatment course for both disorders is for the woman to end the pregnancy through delivery of the placenta; which will stop the more acute effects of the disorders. The timing of this delivery most often results in a premature delivery of human infants, and subsequently offspring from women are often small for gestational age and preterm which increases the risk of neurodevelopmental disorders [5, 6].

Children born to women with HDP like PreE and HELLP have also been reported to have endocrine and cardiovascular morbidities as adolescents and adults [7–9]. There is also increased reports of attention-deficit/hyperactivity disorder (ADHD), intellectual disabilities, autism spectrum disorder and cerebral palsy among children born to women with HDP [10–13]. One study found that as the maternal condition worsened from PreE to severe PreE/HELLP so did the hazard of childhood mental disorders, there is a link between severity of HDP and negative impact on the affected children [14].

Outside of the context of HDP, maternal immune activation during pregnancy also leads to behavioral abnormalities in children [15]. Experimental studies have reported that immune activation during pregnancy is associated with not only HDPs such as PreE and HELLP but also with behavioral abnormalities in offspring from postnatal week 1 up unto postnatal month 3 [16–19]. When the immune system is activated, T cells stimulate an inflammatory response. In both animal models of HELLP and PreE, it has been shown that there is an increase circulating inflammatory cytokines and CD4^+^ T cells, accompanied by hypertension [20–22]. In pregnant women it is well established that maternal immune activation through viral or bacterial infection leads to altered neurobehavioral outcomes, but it is unclear how maternal inflammation stemming from HDP affect the behavioral outcomes of the offspring [23, 24]. Immune activation, specifically an increase in the T cell derived inflammatory Th17 cells, is associated with the pathogenesis of PreE [25, 26]. We have previously reported that blockade of T cell activation with Orencia (Abatacept; a selective co-stimulation modulator which inhibits T cell activation) during pregnancy, prevents the inflammatory and hypertensive response in PreE and HELLP animal models [21, 22]. In the current study we investigated whether offspring born to HDP dams had neurobehavioral disturbances and if suppression of the maternal immune response during pregnancy could reduce or prevent any neurobehavioral disturbances.

## Materials and methods

### Induction of PreE and HELLP in dams and animal grouping

All animals used in this study were offspring born to Sprague Dawley dams (originating from Envigo; Indianapolis, IN). Animals were housed in a temperature-controlled room with a reversed 12:12 light: dark cycle and fed standard rat chow. All procedures were approved by the Institutional Animal Care and Use Committee at the University of Mississippi Medical Center (UMMC) under protocol number 1414 A.

To induce experimental PreE (*n* = 16 dams), mini-osmotic pumps (model 2002, Alzet Scientific, Cupertino, CA) infusing soluble Fms like tyrosine kinase-1 (sFlt-1; 7 µg/kg, R&D Systems, Minneapolis, MN) was mixed with saline and inserted into the intraperitoneal cavity of pregnant dams beginning on gestational day (GD) 12 [27]. Experimental HELLP (*n* = 13 dams) was induced via a similar method, however a combination of sFlt-1 and soluble Endoglin (sEng; 7 and 4.7 µg/kg respectively, R&D Systems) was infused via mini-osmotic pumps [20]. Normal pregnant (NP) dams (*n* = 24) had an abdominal incision made with no mini-osmotic pump insertion. This group represented an uncomplicated pregnancy and served as a normotensive control group. As previous studies have shown that infusion of saline does not alter the cardiovascular, metabolic or immune response in NP dams, pumps were not inserted and only the SHAM surgery was conducted. To attenuate maternal immune activation and hypertension during pregnancy, Orencia ( 2 mg/kg) was infused into a subset of NP (*n* = 10), PreE (*n* = 11) and HELLP (*n* = 9) dams on GD13 via the jugular vein as previously reported [21].

All dams delivered between GD21-22. Immediately after delivery of pups, up to eight pups per litter were removed and cross-fostered with non-study lactating dams with no more than 2 pups from the same litter being placed within the same new litter. The different litters of pups were identified via color markings by using a Sharpie to mark the animals on their back and tail, to identify animals. This mechanism of identification was chosen as there is no pain or a risk of infection compared to ear tagging or tattooing. The initial color marking was performed by an independent investigator to ensure experimenters were blind to pups maternal group. All animals were weaned from foster dams between postnatal days 30–33. At this time they remained in mixed treatment groups and were placed in cages with same-sex cage mates. The following pups were used in the current study: NP male (*n* = 21), NP female (*n* = 21), HELLP male (*n* = 15), HELLP female (*n* = 14), PreE male (*n* = 17), PreE female (*n* = 16), NP + O male (*n* = 15), NP + O female (*n* = 8), HELLP + O male (*n* = 12), HELLP + O female (*n* = 10), PreE + O male (*n* = 11), PreE + O female (*n* = 7).

### Behavior

All animal testing was conducted in the Animal Behavior Core at UMMC and was completed during the animal’s active phase and under low light conditions. Animals were given an hour to acclimate to the testing room unless otherwise noted. For tests that required tracking, the Noldus Observer (Noldus Information Technology, Inc, Leesburg, VA) was used to record and track behavioral testing and a video recorder was used to record sessions that required scoring or validation at a later time period.

#### Social interactions and distance traveled

On postnatal day (PND) 24–26, social interaction and self-initiated play behavior (chasing, following, boxing and pinning) and self-isolation behaviors (self-grooming, self-exploring and time at rest) were measured to assess social play and hyperactivity. Rats were placed in the test arena (60 cm x 30 cm x 40 cm) with a non-familiar conspecific of the same sex and allowed to interact with one another for 20 min. All test sessions were recorded by a camera to allow subsequent scoring which was conducted as previously described [28]. On PND 38–39, open field test was used to assess locomotor activity. Rats were allowed to explore the empty chamber (40 cm x 50 cm x 50 cm) for 20 min in which the distance traveled was captured.

#### Barnes maze

Beginning on PND45, rats were trained in Barnes Maze to assess spatial memory [29]. Rats were placed in a maze surrounded by a set of distinct stationary black and white social cues for 5 days. Rats were placed daily into the goal box (i.e., escape chamber) for a four-minute adaptation period. After four minutes, rats were returned to a holding cage and the goal box was cleaned with 10% ethanol. Rats were then placed directly in the center of the platform and allowed to explore the maze for 5 min. If the goal box was found before time elapsed, that time was automatically recorded and they were allowed to remain in the chamber for 60 s before they were returned to the home cage. If the escape chamber was not located, the subject was returned to the home cage at the end of the trial and the time marked as 301 s.

#### Marble burying

On PND59 anxiety-like behavior was assessed via marble burying. 15 glass marbles were evenly distributed across an empty cage lined with fresh bedding. Rats were allowed 15 min to interact with the marbles before being returned to their home cages and the number of buried marbles was counted. Marbles were considered buried if at least two-thirds were covered by sawdust, with the more marbles that were buried correlating with increased anxiety-like behavior [30]. Cages and marbles were cleaned with 70% ethanol and the sawdust was replaced between subjects.

### Mean arterial pressure (MAP) measurement and organ collection

On PND60 all pups underwent surgery and an indwelling catheter made of V1, V3 and V6 tubing (Scientific Commodities, Lake Havasu City, AZ, USA) was inserted into the carotid artery and then exteriorized and secured at the back of the neck. Rats were allowed to recover overnight and on the following day all rats had their MAP assessed. Briefly, rats were placed in individual restrainers and carotid lines connected to pressure transducers (Cobe III Transducer CDX Sema, Birmingham AL, USA). Following a 30-min acclimation period, MAP was measured for 30-min. After which while under anesthesia, whole blood was collected as were brains. For future studies, the kidney, liver, and spleen were weighed and saved at -80 °C.

### Western blot

After euthanization brains were weighed, dissected and stored at -80 °C. The prefrontal cortex and hippocampus regions were homogenized in Tris lysis buffer containing protease inhibitors, centrifuged and the protein concentration was determined using a bicinchoninic acid protein assay (BCA; ThermoFisher, Rockford, IL) as previously described [31]. Briefly, samples were electrophoresed and transferred onto nitrocellulose membranes using Bio-Rad (Bio-Rad Laboratories, Hercules, CA) gels, membranes, and transfer system, followed by confirmation of transfer via Ponceau-S (Sigma Aldrich, St. Louis, MO) staining. Membranes were blocked with 5% milk buffer, washed in TBST (tris buffered saline with 0.1% tween 20) and allowed to incubate overnight at 4 °C with either mouse anti-neuronal nuclear (NeuN; 1:1000; Catalog #MA5-33103, Bio-legend, San Diego, CA) or Mouse anti-myelin basic protein (MBP; 1:1000; Catalog #808,401, Bio-legend). After washing in TBST, membranes were incubated with 1:1000 anti-mouse HRP-conjugated secondary antibody (Catalog #R1005, Kindle Biosciences, Greenwich, CT), followed by development with KwikQuant Digital enhanced chemiluminescence (Kindle Biosciences). Membranes were imaged with the KwikQuant imager imaging system and protein bands analyzed using Image J software. NeuN and MBP expression were normalized to the amount of total protein present within the sample lane as detected by Ponceau-S [32].

### Statistics

A two-way ANOVA was used to compare differences between groups and sex differences within groups. The ANOVAs were followed by Tukey’s multiple comparisons test or student t-tests were used for additional analysis as needed. For the Barnes Maze analysis, repeated measure two-way ANOVA was performed. Statistical analyses were performed using GraphPad Prism 10. All data were reported as mean ± standard error mean. Values of *p* < 0.05 were considered statistically significant.

## Results

### Maternal Orencia administration increases social play behavior in male offspring from PreE dams and females from HELLP dams

**Fig. 1:**
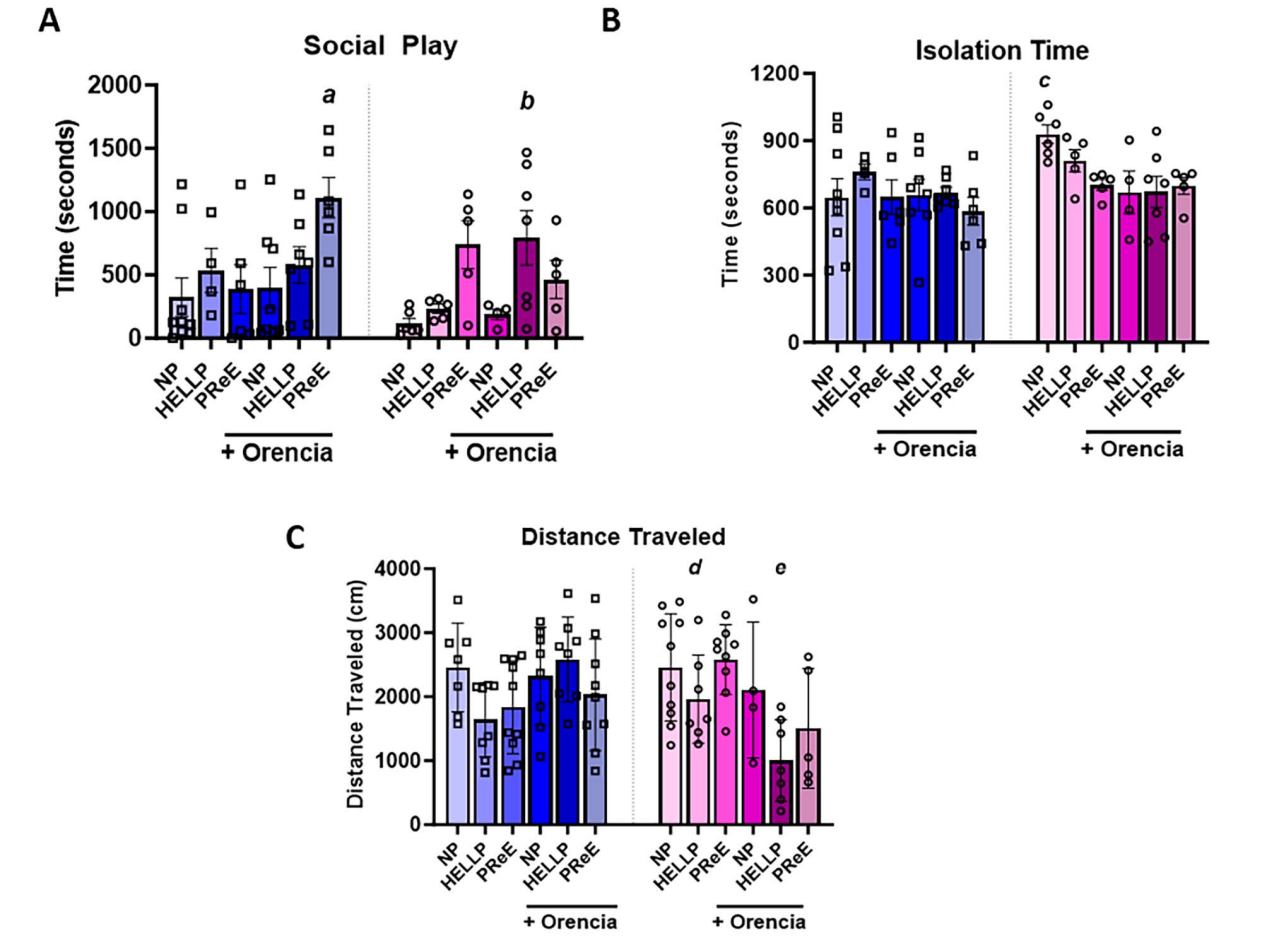
Pups born to dams who had administration of Orencia to otherwise HDP of pregnancy dams showed difference in social play behavior, isolation, and locomotion. Social play behavior separated by sex (**A**), isolation time separated by sex (**B**) and distance travelled separated by sex (**C**) Abbreviations: NP (normal pregnant); PreE (preeclampsia); HELLP (hemolysis elevated liver enzyme low platelet). The open squares denote males and open circles denote females. 1 A legend: *a* denotes *p* < 0.05 between the indicated group and male offspring from NP dams; female offspring from NP, NP + Orencia, and HELLP dams. *b* denotes *p* < 0.05 between the indicated group and female offspring from NP dams. 1B legend: *c* denotes *p* < 0.05 between indicated group and male PreE + Orencia offspring. 1 C legend: *d* denotes *p* < 0.05 between indicated group and NP male and female offspring. *e* denotes *p* < 0.05 between indicated group and male NP + Orencia and HELLP + Orencia offspring and female PreE + Orencia offspring

We examined the effect of HDP with or without immune suppression via Orencia administration on social behavior interactions. The analysis of social play showed a significant group effect (F_2,60_ = 6.63, *p* = 0.003), treatment effect (F_1,60_ = 4.24, *p* = 0.04) and a significant interaction between groups, treatment and sex (F_2,60_ = 5.08, *p* = 0.009, Fig. 1A). Post-hoc analysis showed that males from PreE + Orencia dams spent significantly more time socially playing compared to males from NP dams (*p* = 0.02) and female offspring from NP (*p* = 0.003), NP + Orencia (*p* = 0.03) and HELLP (*p* = 0.03) dams. Female offspring from HELLP + Orencia dams spent more time socially playing relative to female offspring from NP dams (*p* = 0.02). When isolation time (the time spent alone) was analyzed there was not a significant main effect on group (F_2,60_ = 1.32, *p* = 0.28), however there were significant effects on both treatment (F_1,60_ = 5.51, *p* = 0.02) and sex (F_1,60_ = 4.87, *p* = 0.03, Fig. 1B). Post-hoc analysis only indicated that female offspring (928.5 ± 99.9 s) from NP dams spent significantly more time alone vs. male offspring from PreE + Orencia dams (585.4 ± 151.5 s; *p* = 0.02).

### Female offspring from HELLP and HELLP + Orencia dams have reduced locomotion activity

There was not a significant effect on sex (F_1,80_ = 1.69, *p* = 0.2) or treatment (F_1,80_ = 2.1, *p* = 0.15) in locomotor activity assessed by distance traveled. There was a significant main effect on group (F_2,80_ = 3.7, *p* = 0.03, Fig. 1D) and a significant treatment by sex effect (F_1,80_ = 12.44, *p* = 0.0007). Post-hoc analysis indicated that female offspring from HELLP dams traveled significantly less relative to NP offspring (*p* = 0.02 in males and *p* = 0.008 in females). Whereas Orencia treatment to moms further reduced distance traveled in female offspring when compared to male offspring from NP + Orencia (*p* = 0.04) and HELLP + Orencia (*p* = 0.005) dams and female offspring from PreE + Orencia dams (*p* = 0.003).

### Offspring from HDP dams and Orencia treated dams show evidence of impairments in spatial learning and memory

**Fig. 2:**
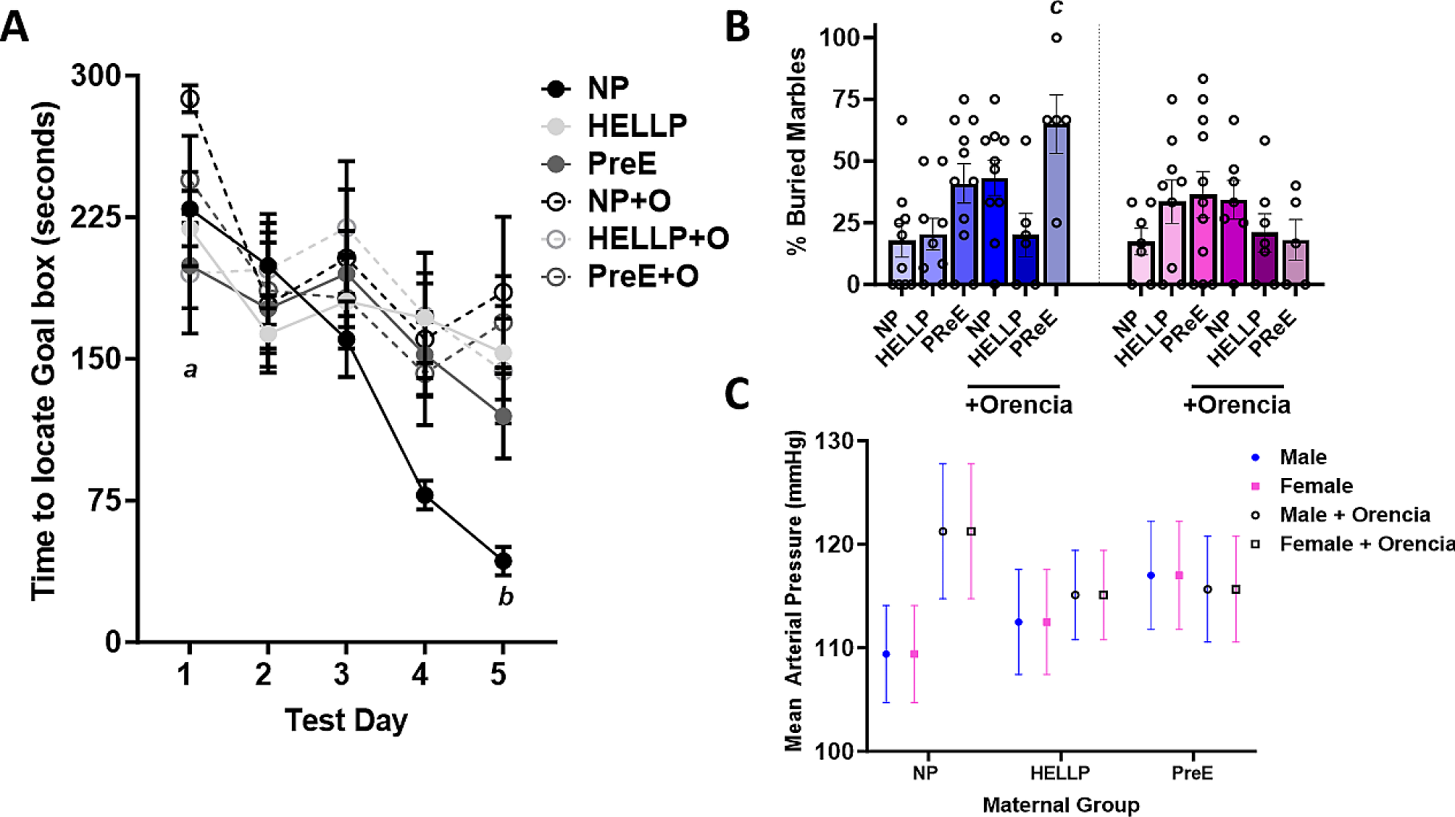
Offspring of HDP regardless of Orencia treatment have spatial learning deficits and anxiety-like behavior. Spatial learning was observed over 5 days in the Barnes Maze (**A**) and anxiety was assessed via the number of marbles buried over a 15-minute period (**B**). There were no significant changes in mean arterial pressure between males and females (**C**). 2 A legend: a denotes that on day 1 of testing offspring from NP + Orencia dams took significantly longer to complete the tasks vs. all other groups except PreE + Orencia. *b* denotes that on day 5 of testing NP offspring completed the task significantly faster relative to all other groups. 2B legend: *c* denotes *p* < 0.05 between the indicated group and NP male, NP female and HELLP male offspring

Because sex did not have a significant effect on performance in the Barnes Maze (Data Supplement), sexes were collapsed for the subsequent analyses. There was not a main effect on group (F_5,89_ = 1.9, *p* = 0.10) in the Barnes Maze, however significant main effects were present when the days (F_4,356_ = 15.92, *p* < 0.0001), subjects (F_89, 356_ = 2.07, *p* < 0.0001) and the interaction between groups and days (F_20,356_ = 1.69, *p* = 0.03) were examined (Fig. 2A). On day 1 of testing offspring from NP + Orencia dams took significantly longer (*p* < 0.05) to complete the task compared to every group except for offspring from PreE + Orencia dams, whereas by day 5 of the test offspring from NP dams completed the task significantly faster relative to offspring from all other groups (*p* = 0.002). Post-hoc analysis found that offspring from NP (*p* < 0.0001), HELLP (*p* = 0.01), NP + Orencia (*p* = 0.03) and PreE + Orencia (*p* = 0.03) dams demonstrated significant improvement over the testing period relative to Day 1.

### Offspring from PreE, PreE+ Orencia and NP + Orencia dams have increased anxiety

When anxiety was assessed via marble burying there were no significant effects on sex (F_1,85_ = 2.48, *p* = 0.12) but there was a significant main effect between groups (F_5,85_ = 2.92, *p* = 0.02) where offspring from NP dams buried significantly less marbles (i.e. less anxiety) relative to offspring from PreE (*p* = 0.01), NP + Orencia (*p* = 0.003) and PreE + Orencia dams (*p* = 0.02, Fig. 2B). There was a significant interaction between sex and group (F_2,85_ = 3.59, *p* = 0.03) where male offspring from PreE + Orencia dams buried significantly more marbles relative to males from NP (*p* = 0.02) and HELLP (*p* = 0.04) dams and when compared to female offspring from NP dams (*p* = 0.04). There was also not a significant effect of maternal Orencia treatment offspring marble burying (F_1,91_  =  1.44, *p* = 0.23).

### No changes in blood pressure or protein expression

**Fig. 3:**
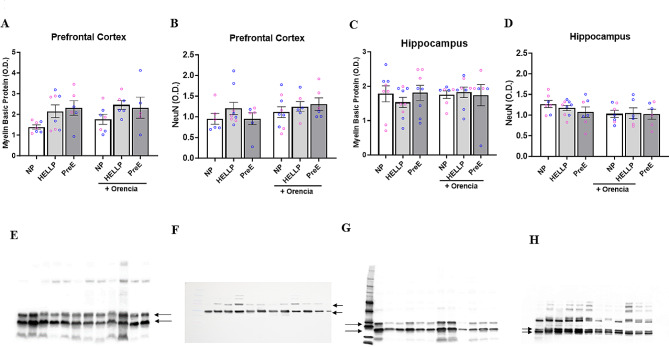
No difference in MBP and NeuN expression in the hippocampus and prefrontal cortex between groups. Representative western blot of homogenates (30 µg) from the prefrontal cortex (**A, E**) and (**B, F**) and the hippocampus region **(C, G)** and **(D, H)** with expression of Myelin Basic Protein and NeuN respectively depicting the characteristic bands at 20 kDa for MBP and 45–55 kDa for NeuN, and bar graphs showing the relationship between groups and sex within each respective brain region. Data are represented as mean ± SEM; *n* = 3–5/group/sex

MAP was assessed to determine if offspring from hypertensive dams were also susceptible to hypertension. There were no significant differences between groups due to sex (F_1,106_  =  0, *p* = 1), group (F_2,106_  =  0.19, *p* = 0.82) or Orencia treatment (F_2,106_ = 1.84, *p* = 0.18; Fig. 2C). Finally, neural expression of MBP and NeuN were evaluated as both have a role in the neural connections associated with learning and memory. There was no significant main effect on sex in either MBP prefrontal cortex (F_1,27_ = 1.02, *p* = 0.32) or hippocampus (F_1,34_ = 1.21, *p* = 0.28) protein expression or with NeuN prefrontal cortex (F_1,30_ = 0.83, *p* = 0.37) or hippocampus (F_1,30_ = 3.55, *p* = 0.07) expression, therefore sex data was collapsed within groups. In the prefrontal cortex, there was a significant difference between groups in MBP (F_2,33_ = 4.29, *p* = 0.02, Fig. 3A, E) with post-hoc analysis indicating no specific group differences. There were no significant differences in NeuN protein expression (F_2,36_ = 0.93, *p* = 0.40, Fig. 3B, F). Similar results were seen in the hippocampal expression of MBP (F_2,40_ = 0.16, *p* = 0.85, Fig. 3C, G) and NeuN (F_2,36_ = 0.5, *p* = 0.61, Fig. 3D, H). In no cases were there any significant differences in protein expression due to treatment of Orencia (Data Supplement).

## Discussion

The current study evaluated if offspring born to hypertensive dams treated with or without an immune suppressant were susceptible to behavioral abnormalities. PreE and/or HELLP have been reported to have over activation of the immune system which is believed to contribute to the pathophysiology of these pregnancy disorders [33, 34]. Clinical studies have identified an association among children born to women with HDP in which the children present with mood disorders (anxiety and depression), autism, cognitive deficits and ADHD [10, 35, 36]. To determine if suppression of immune activation improves behavior among adolescent offspring, pregnant dams were treated with Orencia in an effort to prevent CD4^+^ T cell activation. Important to this growing body of work are the new data that we provide demonstrating sex specific differences in offspring from both PreE and HELLP dams and further insight into the effects of Orencia administration during pregnancy.

Social interactions and play behavior in rats during the adolescent period are measurements of socio-cognitive skills that are relatable to the development of social behavior in humans [37, 38], therefore we wanted to evaluate play behavior in the current study. Offspring from PreE and HELLP dams responded differently in the behavioral challenges, indicating that the degree of maternal hypertension and immune activation *in utero* impacts the behavior of offspring. Immune suppression in HDP led to evidence of increased social play. Overall, these offspring showed increased time socially interacting and playing, which did not directly translate to an increase in distance traveled. Again supporting the idea of hyperactivity or enhanced social play among some groups. Opposite effects were seen in offspring from normotensive rats subjected to immune suppression; these rats preferred to spend more time in isolation while there was not a great effect on their distance traveled. There was also an increase in marble burying activity among male PreE + Orencia offspring. The increased number of buried marbles has been interpreted to be both an indicator of increased anxiety-like behavior as well as the repetitive behavior that is seen in individuals along the autism spectrum or with obsessive-compulsive disorders [39]. To determine if these behavioral results are more in line with the repetitive behavior seen in neurodevelopmental disorders vs. anxiety-like behavior, additional behavioral studies of these disorders would need to be conducted.

Preclinical studies have shown behavioral and sex differences in offspring of maternal immune activation models where male offspring were more likely to exhibit anxiety and deficits in spatial working memory relative to female offspring [40, 41]. Impairments in spatial learning and memory have also been reported by others, as offspring from the L-NAME model of PreE have impairments in spatial learning and memory [42, 43]. Immune suppression in the normotensive dams led to offspring having worse outcomes on day 1 of the spatial learning test. By the end of the test, offspring fromPreE dam had no significant improvement of spatial learning from baseline. In addition, regardless of hypertension or immune suppression all offspring had learning impairments compared to normotensive offspring. Immune suppression decreased anxiety-like behavior in offspring from HDP rats. The immune suppression during pregnancy has a similar effect on the maternal presentation of anxiety-like behavior [27, 44].

Abnormal myelination in both clinical and pre-clinical studies has been correlated with cognitive deficits in frontal-temporal and hippocampal regions of the brain in psychotic disorders, ADHD, and Alzheimer’s [45–47]. Studies have also found that increased MBP and NeuN expression among offspring exposed to *in utero* inflammation was found to be associated with improvements in learning and memory [48, 49]. Despite these reports, MBP, responsible for myelinating nerves, and NeuN were not increased in either the prefrontal cortex or the hippocampus in the current study. It has been reported that the peak time for MBP expression in rodents is PND 20, after which expression stabilizes [50], if this is the case it might explain the lack of differences among our groups. Interestingly, PreE offspring (from the L-NAME model) that expressed impairments in spatial learning and memory, also had changes in cortical, hippocampal and striatal NeuN expression when assessed via immunohistochemistry [42, 51]. The findings of no change in blood pressure among offspring from HDP dams in the current study is similar to another study with similar findings when offspring from PreE dams were examined [52].

There are several different animal models of maternal immune activation and of HDP, many of which utilize different methods to induce hypertension and/or immune activation. Despite the difference in methodologies, our results are similar to what has been reported among male offspring. In an arginine vasopressin model of PreE, male offspring of PreE dams exhibited increased social behavior in the three-chamber social preference task relative to control offspring, without there being a significant difference in distance travelled between the groups [53]. Our study is not without limitations, firstly even though it has previously been demonstrated that these animal models have hypertension and inflammation during pregnancy we did not directly evaluate that in the dams that birthed the offspring used in the current study [20, 54]. We also did not measure the immune profile of these offspring as immune activation during pregnancy can be correlated with neurodevelopmental outcome of offspring [55]. Future studies need to include a variety of test to assess learning and memory to further elucidate the different roles that the prefrontal cortex and hippocampus have these offspring and to determine if there are sex differences.

Clinically Orencia is only administered during pregnancy when the maternal benefit outweighs the potential risk to the fetus. However, despite this classification clinical studies have found no association between Orencia exposure *in utero* and fetal death or the development of congenital anomalies [56]. The findings from the current study indicate that despite the previous reports of maternal benefit during pregnancy [21, 22], maternal immune suppression as a result of Orencia may have negative effects on offspring. This is especially true in instances where there was likely not a robust immune response such as in NP rats. Immune suppression in the absence of an immune response or via CD4^+^ regulatory T cell depletion has been linked with increased anxiety-like behavior, depression and alterations in social behavior [57, 58] and might in fact be one reason why marble burying is increased in our control NP + Orencia offspring.

Perhaps due in part to the lower incidence of HELLP, the majority of clinical studies evaluating the long-term effects of HDP on offspring do not distinguish between mothers diagnosed with PreE vs. HELLP. There were also distinct differences between male and female offspring both in their behavior and in the degree and rate of brain maturation, emphasizing the increased need for additional studies to delineate sex differences. The results from the current study add to the growing body of evidence indicating that HELLP is a different maternal indication rather than a subtype of PreE [59, 60], and highlight the importance of evaluating the classifications of HDP when assessing neurodevelopment [61].

## Perspectives and significance

This study was able to demonstrate that Orencia administration during a preeclamptic and HELLP pregnancy led to different effects on social interactions and locomotion. These different effects also were sex dependent. Overall Orencia did not improve behavior in offspring from HDP rats, it is possible that the timing of immune suppression during pregnancy had detrimental effects on the offspring (as seen in NP + Orencia offspring) rather than protective effects. Previous studies have also reported that when Orencia is administered during pregnancy and animals are evaluated during pregnancy, babies from HELLP + Orencia dams are smaller relative to untreated dams [21]. Whether the offspring was from an HDP dam or Orencia treated dam, there was evidence of learning impairments independent of sex. This study adds to the growing body of knowledge indicating that PreE and HELLP Syndrome may have a differential effect on offspring behavioral development which is further complicated by sex and possible in utero exposure to immune therapeutics.

### Electronic supplementary material

Below is the link to the electronic supplementary material.

Supplementary Material 1

Supplementary Material 2

## Data Availability

All data generated or analyzed during the study are included in this published article and its supplementary information files.
