# Supplementary material for: Maternal immune suppression during pregnancy does not prevent abnormal behavior in offspring
Source: Biol Sex Differ. 2024 Mar 26;15:27. doi: 10.1186/s13293-024-00600-8 (PMC10967052; doi:10.1186/s13293-024-00600-8)
Supplement: Supplementary file 1 — Supplementary Material 1 [file 13293_2024_600_MOESM1_ESM.docx]

**Data Supplement.**

**Table 1.** P values between male and female offspring throughout the Barnes Maze.

| **P value** | **Day 1** | **Day 2** | **Day 3** | **Day 4** | **Day 5** |
| --- | --- | --- | --- | --- | --- |
|  | 0.22 | 0.55 | 0.22 | 0.90 | 0.29 |

**Table 2.** Orencia does not significantly affect protein expression.

|  | **F (DFn, DFd)** | **P value** |
| --- | --- | --- |
| **Effects of Orencia on:**  Hippocampal MBP  Hippocampal NeuN  Prefrontal cortex MBP  Prefrontal cortex NeuN | F (1,40) = 0.17  F (1,36) = 2.81  F (1,33) = 0.89  F (1,36) = 2.47 | 0.68  0.10  0.35  0.12 |
