# Supplementary figures and images for: Maternal immune suppression during pregnancy does not prevent abnormal behavior in offspring
Source: Biol Sex Differ. 2024 Mar 26;15:27. doi: 10.1186/s13293-024-00600-8 (PMC10967052; doi:10.1186/s13293-024-00600-8)

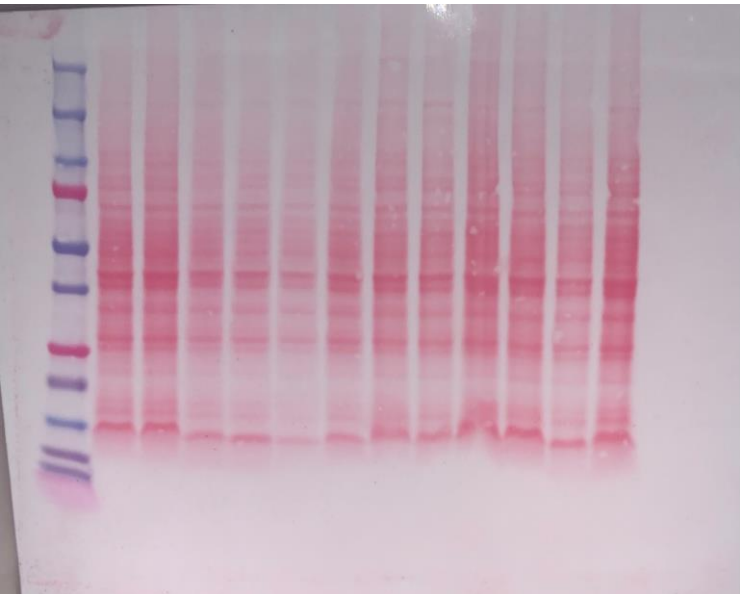

MBP Prefrontal Cortex

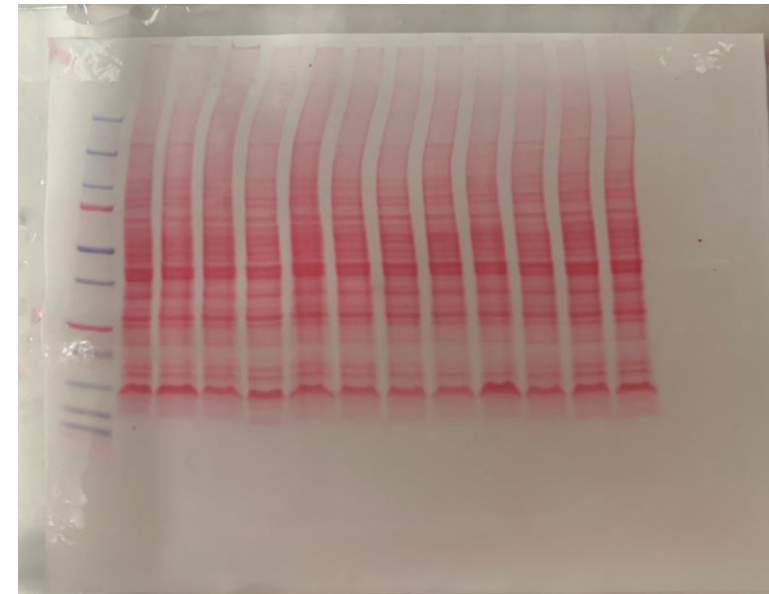

NEUN Prefrontal Cortex

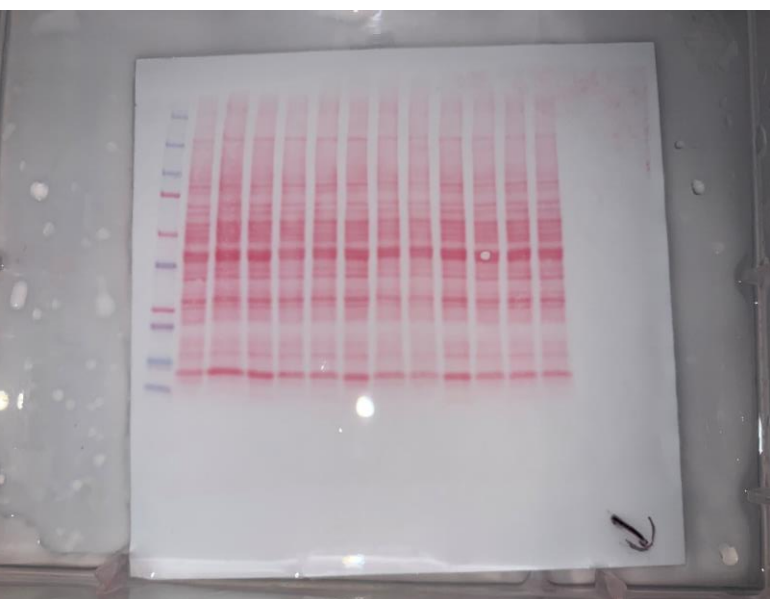

MBP Hippocampus

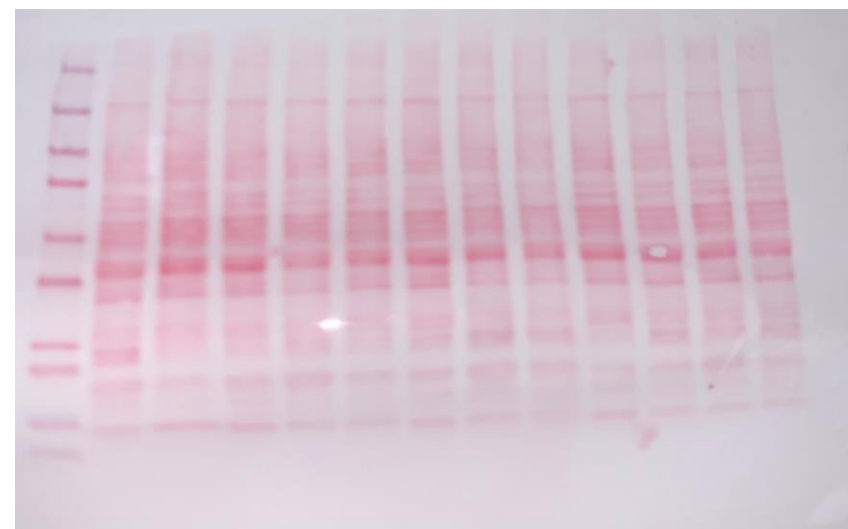

NEUN Hippocampus

Supplement: Supplementary file 2 — Supplementary Material 2 [file 13293_2024_600_MOESM2_ESM.pdf]
